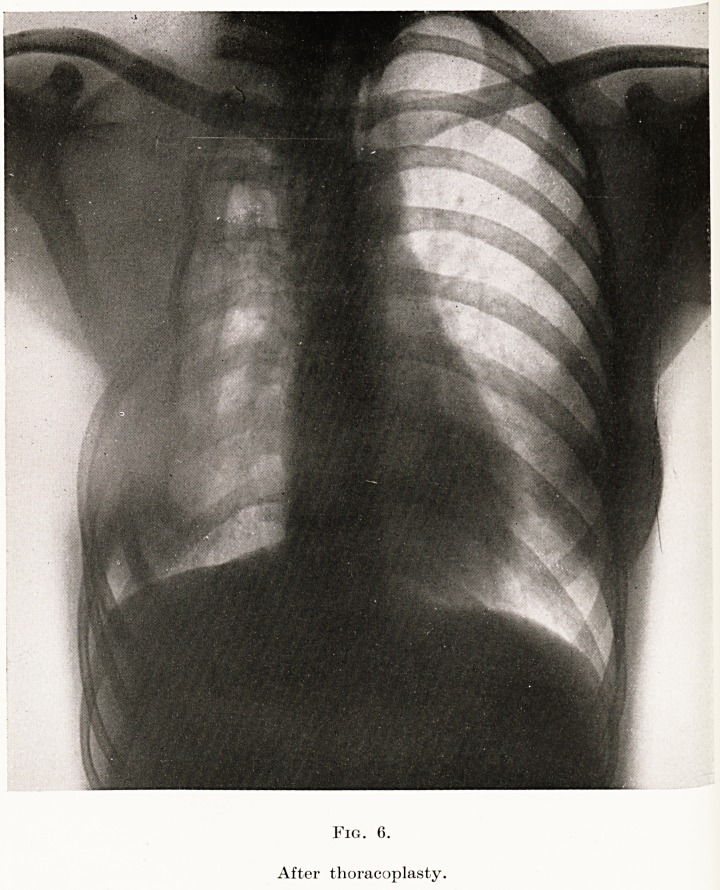# Some Tuberculous Conditions
*Presidential Address, delivered before the Bath and Bristol Branch of the British Medical Association at Bristol on Wednesday, 28th September, 1938.


**Published:** 1938

**Authors:** Hubert Chitty

**Affiliations:** Surgeon to the Bristol Royal Infirmary and to Winford Orthopædic Hospital; Consulting Surgeon to the Royal Hospital for Sick Children and Women, Bristol


### SOME TUBERCULOUS CONDITIONS.*

BY

Hubert Chitty, M.S., F.R.C.S.,

*Surgeon to the Bristol Royal Infirmary and to Winford Orthopædic Hospital;*

*Consulting Surgeon to the Royal Hospital for Sick Children and Women, Bristol.*

---

During the present century there has been a truly remarkable diminution in the incidence of tuberculous disease. There can be no doubt that this has been mainly brought about by preventive measures. It is, in fact, one of the outstanding triumphs of preventive medicine.

The phthisical patient is no longer given a bottle of medicine and allowed to return home carrying infection to his family and associates. He is either isolated in a sanatorium or is taught how to live at home without endangering the lives of those with whom he is in contact, and is kept under supervision throughout the course of his illness.

From the point of view of the patient and the public the results have been excellent; from that of the medical student, however, they have been much less satisfactory. Tuberculous subjects do, of course, still come up to the Out-Patient Departments of general hospitals for investigation; but, once their ailments

* Presidential Address, delivered before the Bath and Bristol Branch of the British Medical Association at Bristol on Wednesday, 28th September, 1938.

have been diagnosed, they are seen there no more, and many students are getting qualified who have had little experience in the diagnosis of tuberculous conditions and none in their treatment.

During the same period considerable changes have also taken place in the treatment of tuberculosis of both the pulmonary and the so-called "surgical" varieties. At the present time the surgeon and the physician co-operate so closely in the treatment of all types of the disease that it is no longer easy to draw any distinction between what is medical and what surgical. In my younger days it was rare indeed for a surgeon to be called in to see a case of pulmonary tuberculosis. Should a child suffering from tuberculous disease of a bone or joint be admitted to hospital, however, the surgeon boldly attacked the case without any preliminary medical treatment. The results were, I regret to say, by no means satisfactory. Now and then an excision or erasion of a joint met with success, but many of the operation wounds broke down and became secondarily infected, with eventual loss of limb or life; while quite a large number of the patients subsequently developed miliary tuberculosis.

It was during the first decade of the present century that the treatment of non-pulmonary tuberculosis in open-air hospitals and by other than operative methods became really popular. There was such a striking improvement in the results, together with a great diminution in the mortality, that for a time the operative treatment of tuberculous bones and joints almost disappeared. Non-operative methods still reign supreme, but of recent years certain operative measures have been introduced which have lessened the period of invalidism and have considerably improved the final results. Surgical methods as applied to the treatment of pulmonary tuberculosis are a comparatively recent development. The field is limited, but the results in suitable cases are undoubtedly very satisfactory. In the present paper I propose to confine myself to some observations upon tuberculosis of the hip, the spine and the lungs.

### TUBERCULOUS DISEASE OF THE HIP.

The vast majority of cases occur in children during the first ten years of life. The condition seems particularly rare in adults. Once the disease is *well established* there is little difficulty in effecting a diagnosis. The text-book description and a good skiagram should leave little doubt as to the nature of the trouble. It is in *the very early case* that doubts not infrequently arise ; and it is at this stage, when bone changes are few, that treatment should be instituted if the best results are to be obtained. Delay must increase the period of invalidism and mar the final result.

The symptoms are fairly constant. In almost every case a child is brought to see one on account of a limp, which has usually been present for some weeks. There may or may not have been some pain in this early stage. If present it is much more often referred to the knee than to the hip, and many a time have I seen cases in which an inexperienced house surgeon has examined and radiographed the knee when the real trouble has been in the hip joint. Mistakes of this kind are much less likely to occur if one insists upon stripping every child who has a limp. As the child walks it will then be seen that there is some flexion at the hip joint, a sign which is absent in cases of painful knee. When the child is examined supine the hip flexion is often masked by lordosis, but if this is overcome by persuading the child to put its sound knee to its mouth the flexion immediately becomes apparent. In addition to the flexion there is present some asymmetry of the two limbs. In early cases the thigh is usually abducted and outwardly rotated. The child should then be turned upon its face, and first the sound and then the affected limb be lifted off the couch by grasping the child's ankle. Loss of hyper-extension is very readily demonstrated by this manœuvre. Another early and very constant sign is wasting of the thigh muscles.

*X-ray Appearances*

The absence of any bone changes does not mean that the case is non-tuberculous. The primary lesion may be synovial. The most constant changes are :—

1. Loss of symmetry: the affected hip being abducted and rotated out.

2. Decalcification of the bony structures forming the joint. Do not forget that the disease may start in the acetabulum and that the innominate bone may be the first to show rarefaction.

3. Loss of joint space.

4. Blurring of the bony outlines, suggestive of a poor skiagram. These early changes may easily be overlooked unless both hips are taken on the same plate, a precaution which should NEVER BE NEGLECTED.

There are a few not uncommon conditions which mimic an early tuberculous hip. Coxa plana and slipped epiphysis should be readily differentiated by means of a skiagram. A limp, together with flexion of the hip, may be due to an inflamed inguinal gland or to psoas spasm caused by retrocaecal appendicitis or pelvic inflammation. A careful physical examination should serve to differentiate these. Poliomyelitis may give rise to a limp, wasting of the thigh muscles and decalcification of bone: but the history, the presence of free and painless hip movements and probably evidence of muscular wasting elsewhere should save one from making a mistake.

The condition which I find most difficult to differentiate is a temporary synovitis due to some other cause. The history is generally a short one; the pain and limp may have followed a cold or sore throat and there is no muscular wasting. In a case of this kind one must withhold judgment. Treat the case as a possible tuberculous infection; put the child to bed for a week or two and then re-examine. The rest will have made little difference to a tuberculous case, but a synovitis due to other causes will generally have cleared up completely and left no signs of any disability.

*Treatment.*

This consists first in measures designed to increase the general powers of resistance. Of these the chief are complete rest in bed, good food, sunlight and nursing day and night in the open air. The value of the stimulant action of air in movement striking upon the exposed body is not as much appreciated as it should be. The addition of this one factor to the treatment of a child who has been nursed in a hospital ward makes a truly remarkable difference to its well-being. The appetite increases by leaps and bounds, the cheeks become rosy, and the whole temperament of the child alters.

Local treatment consists in traction upon the limb till any deformity has been corrected, followed by complete immobilization till healing is complete. Different methods of fixation are adopted at different hospitals. I do not propose to discuss their merits and demerits in the present paper.

During the treatment of these cases the complication most likely to arise is abscess-formation. In this event no immediate operation is called for as quite a large number of abscesses are spontaneously absorbed. But should the skin threaten to become involved the abscess should be aspirated through a good thickness of healthy tissue. The aspiration should be repeated as often as necessary. In spite of treatment sinuses will develop in a few cases and in them the most meticulous care must be taken to avoid mixed infection. This is, of course, a disastrous occurrence. At the best it will seriously prolong the period of invalidism; at the worst it may be followed by loss of life or limb.

One of the most difficult problems to decide is: "How long must one continue treatment?" Two definite rules can be laid down upon this point. First, all pain and muscle-spasm must have been absent for several months. Secondly, X-ray examination must show not only that the disease has ceased to spread, but that recalcification is taking place, as evidenced by increased bone density and improved definition. Even when satisfied upon these points one must proceed with great caution. At first the child should be allowed up for a short time each day with the hip fixed in plaster. If all goes well the "up" time is slowly increased, and eventually some weight-bearing is allowed, still with the hip fixed in plaster. Should there be any sign of a flare-up, such as a return of pain in the hip or knee, back the child must go to bed for some further months of rest and immobilization.

PLATE XIII

Fig. 1.

Extra-articular graft.

### PLATE XIV

Fig. 2.

Bony ankylosis of both hip-joints.

Fig. 3.

Operative production of Pseudarthrosis.

A mobile hip joint is never to be expected. What one hopes for is bony ankylosis in a good position. In a great many cases the ankylosis is fibrous and this may lead to bitter disappointment. So often a child is discharged from hospital with the hip in excellent position only to return later with flexion, adduction and internal rotation, and perhaps by that time bony ankylosis in this faulty position. If such should be the case an osteotomy is imperative. Without this treatment the patient will go through life with a bad limp and wearing a high boot. An osteotomy, on the other hand, will correct any apparent shortening and will permit the patient to walk with scarcely any perceptible limp. It is to avoid this disappointing development of late deformity and also to expedite healing of the tuberculous lesion that the operation of extra-articular arthrodesis has become popular of late years. As usually practised nowadays, a large bone graft is turned down from the ilium and inserted into a slot made in the great trochanter. The first skiagram (Fig. 1) will show the result of such an operation. The second skiagram (Fig. 2) shows a case in which bony ankylosis of both hip joints has taken place. In this instance the child was unable to get about until I had formed a false joint by dividing the neck of the femur and inserting between the raw bone surfaces a sheet of fascia lata. She now has about 45 degrees of active flexion and is able to walk with remarkable freedom. (Fig. 3.)

### TUBERCULOUS DISEASE OF THE SPINE.

This is much the commonest of all tuberculous infections of the bones and joints. It is quite common in adults as well as in children. The dorsal region is most frequently affected and it is the site most difficult to treat. The reasons for this are :—

1. That the disease usually commences in the front of the vertebral bodies, and that the spine is kyphotic in the dorsal region : this leads to crushing of the diseased portion of the bone and to an early angulation of the spine ;

2. In the dorsal region the spinal canal is smaller than elsewhere so that any swelling due to abscess formation or granulation tissue very readily causes pressure upon the cord.

*Diagnosis.*

The commonest symptom is persistent pain usually referred to the back. It is increased by jarring and relieved by recumbency. At times the pain is referred to the chest, the abdomen or the limbs. Now and again a case presents itself in which there has been no complaint of pain at all and which comes up on account of deformity, of abscess formation, or even of paraplegia.

When first seen some deformity is not uncommonly already present. Usually there is local rigidity of the spine and pain is elicited when the affected part is jarred. In early cases the diagnosis is frequently in doubt till a good skiagram has been obtained. An early lesion is much more easily detected in a lateral than in an antero-posterior skiagram. In the latter the earliest sign is a narrowing of the intervertebral disc.

Very many cases have been treated for months as sciatica or lumbago, and I would urge my fellow medical men not to diagnose these diseases till every effort has been made to exclude other possible causes of pain. Occasionally referred pain has given rise to suspicion of some abdominal complaint such as cholecystitis, appendicitis or gastric ulcer. Scoliosis should not cause confusion, as tuberculosis gives rise to angulation rather than to rotation. Spondylitis deformans and malignant disease can be differentiated skiagraphically.

*Treatment.*

In the case of children this consists in recumbency till the disease is not only arrested but healed. This may mean several years; never less than one. No external splint will prevent the diseased vertebrae being crushed together if the patient is in an upright position. With disease in the lumbar and lower dorsal regions I treat my patients prone in a plaster bed. When it occurs above this level the patient is better nursed supine, in a plaster bed which should extend upwards to include the head and neck.

In the case of adults the period of invalidism can be considerably shortened if the diseased vertebrae are fixed together by a bone graft: but this operation should be deferred till the disease has become quiescent. I always treat my adult patients by a few months recumbency in the open air till their resistance has been raised and all pain has disappeared, and then insert an Albee bone graft. The operation is not a serious one. With a good assistant it can be carried out in half to three quarters of an hour, and is not followed by any shock. Some three months later it is generally possible to allow the patient to sit up, and the majority are able to return to work about a twelvemonth later.

The chief complications likely to arise in cases of tuberculous spine are:—

1. Paraplegia. This usually clears up with rest. Should it develop when the patient is supine it will often disappear if the patient is nursed prone and *vice versa.* The most troublesome cases are those which develop when the tuberculous disease is of long standing and apparently quiescent. In many such there is present an abscess the pus in which has become inspissated or calcified. (Fig. 4). Such an abscess is commonly situated in the posterior mediastinum and needs to be evacuated before the paralysis will clear up. To effect this, the level of the abscess must be accurately determined and the corresponding transverse process and rib base cut away. This allows access to be obtained to the front of the vertebra, when the pus may be scooped out. The operation is generally very successful. (Fig. 5.)

2. Abscesses in other situations may need aspiration, but the majority are gradually absorbed.

### PULMONARY TUBERCULOSIS.

In my early days when I still had plenty of time on my hands I became interested in the treatment of unilateral pulmonary tuberculosis by the induction of artificial pneumothorax. Tuberculosis in other parts of the body had been found to respond so satisfactorily to rest that it seemed sensible to adopt the same principle in the treatment of pulmonary cases. Dr. Campbell Faill had recently come to Bristol, and together we carried out the first series of cases in these parts. Of course this mode of treatment is extensively employed at all sanatoria at the present day.

There are cases in which generalized adhesions render the induction of an artificial pneumothorax impossible and others in which localized adhesions render it unsatisfactory by preventing the collapse

PLATE XV

Fig. 4.

Paravertebral abscess ("butterfly"). Before operation.

Fig. 5.

Same case after operation.

### PLATE XVI

Fig. 6.

After thoracoplasty.

of cavities. In such a case the patient still suffers from persistent cough and expectoration and from the effects of toxic absorption. Where adhesions are small they may be divided by means of a cautery or by diathermy. This operation is not devoid of risk, because there may be an extension of a cavity into the adhesion itself, and it may inadvertently be opened when the adhesion is divided. This in turn may lead to the production of an empyema, possibly with fatal results. It is well, therefore, to limit oneself to small adhesions and to divide them as close as possible to the chest wall.

Where basal adhesions are present a phrenic evulsion, by paralysing the diaphragm, allows it to rise and so to counteract the effect of the adhesions. The operation is a simple one which is carried out under local anæsthesia. Perhaps on this account, it seems to be very popular with physicians. The same operation is also of value at times in putting a stop to recurrent hæmoptysis.

When extensive adhesions are present more radical methods are adopted for putting the lung at rest. Of these the two most commonly employed are apicolysis and thoracoplasty. In apicolysis the parietal pleura is freed from the upper part of the chest wall to enable the upper part of the lung to collapse. The space thus formed is filled with paraffin wax. It is an excellent operation, but has two drawbacks ; the first that the foreign body may gradually be extruded ; the second that it may give rise to septic infection.

For my own part I have a preference for the operation of thoracoplasty. This consists in the removal of large portions of the fixed ribs, from their spinal attachments outwards and including their angles. This enables the chest wall to fall inwards. If it is desired to collapse the upper part of the lung only, it suffices to attack the upper five or six ribs. Where a more extensive collapse is required the operation is carried out in two stages, the upper five ribs being removed two or three weeks before the remainder. The operation produces remarkably little constitutional disturbance when one considers its magnitude. The results are excellent, and it is surprising how little deformity is noticeable when the patient is clothed. (Fig. 6.)

## Figures and Tables

**Fig. 1. f1:**
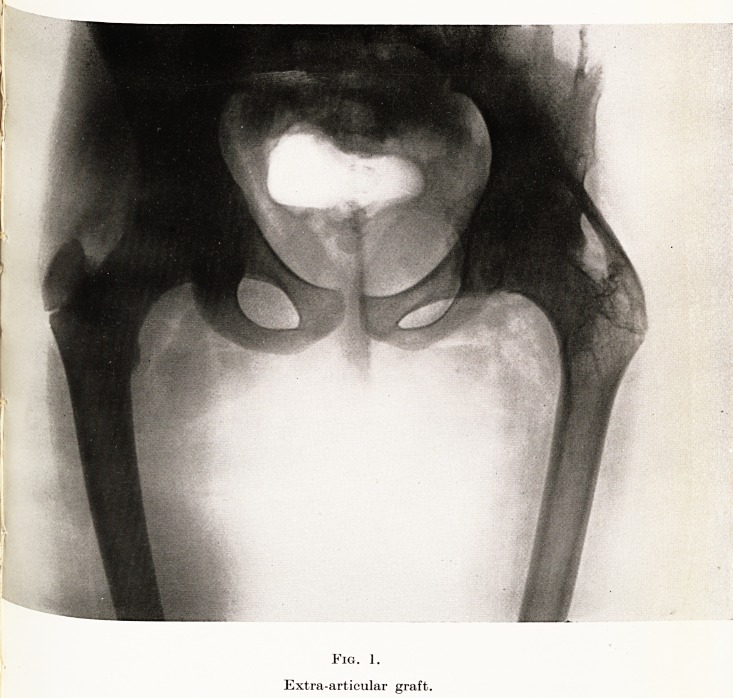


**Fig. 2. f2:**
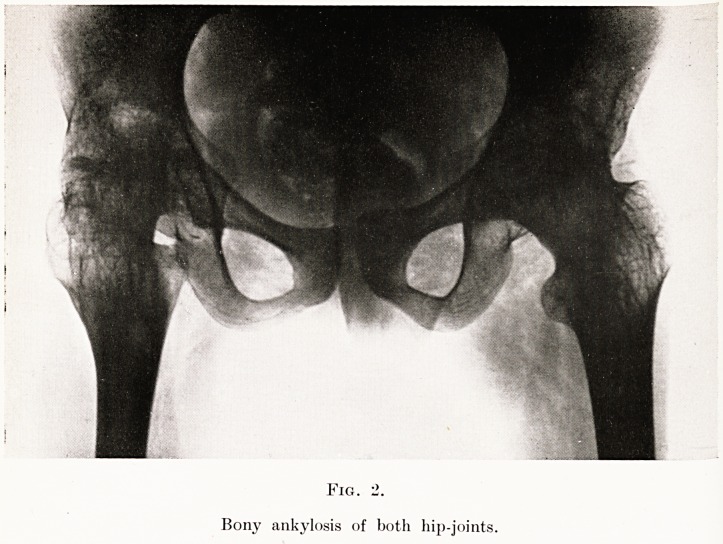


**Fig. 3. f3:**
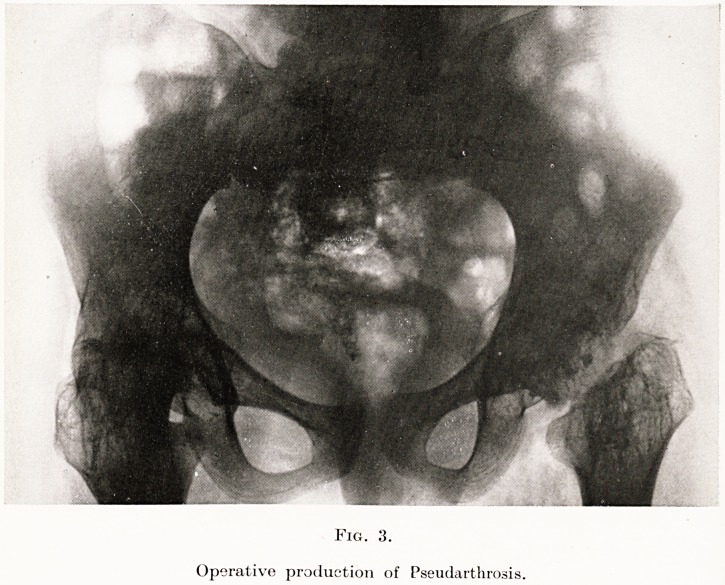


**Fig. 4. f4:**
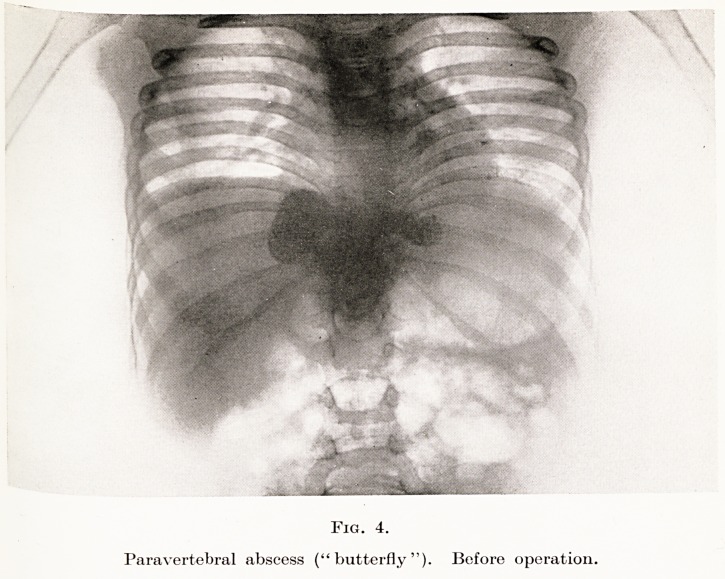


**Fig. 5. f5:**
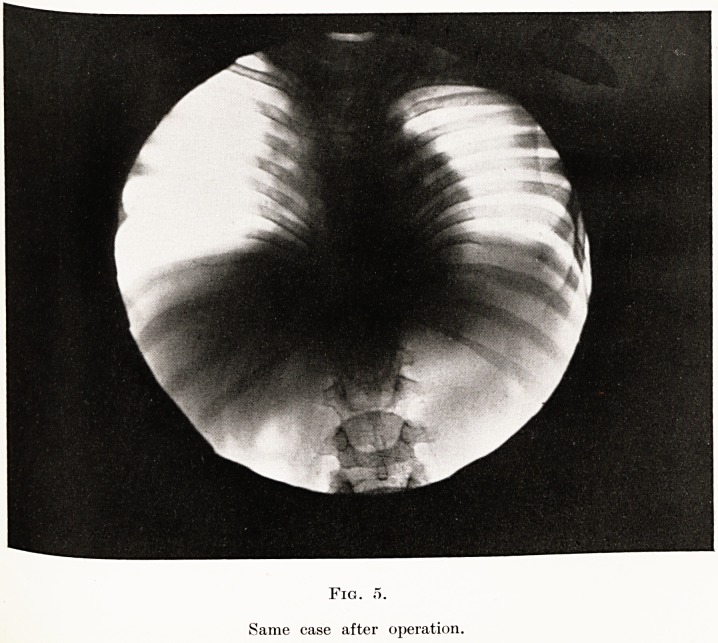


**Fig. 6. f6:**